# How non-functioning pituitary adenomas can affect health-related quality of life: a conceptual model and literature review

**DOI:** 10.1007/s11102-017-0860-4

**Published:** 2018-01-04

**Authors:** Cornelie D. Andela, Daniel J. Lobatto, Alberto M. Pereira, Wouter R. van Furth, Nienke R. Biermasz

**Affiliations:** 1 Department of Medicine, Division of Endocrinology, and Center for Endocrine Tumors, C7-Q, Leiden University Medical Center, P.O. Box 9600, 2300 RC Leiden, The Netherlands; 2 Department of Neurosurgery, Leiden University Medical Center, Leiden, The Netherlands

**Keywords:** Quality of life, QoL, Health-related quality of life, HR-QoL, Well-being, Patient reported outcome, Pituitary adenoma, Non-functioning pituitary adenoma, Wilson–Cleary model

## Abstract

After treatment for a non-functioning pituitary adenoma (NFA) health-related quality of life (HR-QoL) improves considerably. However, the literature about the normalization of HR-QoL after treatment is inconclusive. Some researchers described a persistently decreased HR-QoL compared to reference data, while others did not. Considering this variety in observed HR-QoL outcomes, the aim of the present review was to provide a literature overview of health outcomes in patients with a NFA, using a conceptual HR-QoL model. A concrete conceptualization of the health outcomes of patients with a NFA can be helpful to understand the observed variety in HR-QoL outcomes and to improve clinical care and guidance of these patients. For this conceptualization, the Wilson and Cleary model was used. This model has a biopsychosocial character and has been validated in several patient populations. In the present review, health outcomes of patients with a NFA were described at each stage of the model e.g. biological and physiological variables, symptom status, functional status, general health perceptions and overall HR-QoL. The Wilson–Cleary model elucidates that elements at each stage of the model can contribute to the impairment in HR-QoL of patients with a NFA, which explains the reported variety in the literature. Furthermore, by applying the model, potential interventions targeting these elements can be identified. While optimal biomedical treatment has always been the focus, it is clearly not sufficient for good HR-QoL in patients with a NFA. Further improvement of HR-QoL should be supported by a pituitary specific care trajectory, including psychosocial care (e.g. self-management training), to beneficially affect characteristics of the patient and the (healthcare) environment, with the utmost goal to optimize HR-QoL in patients after treatment.

## Background

**Fig. 1:**
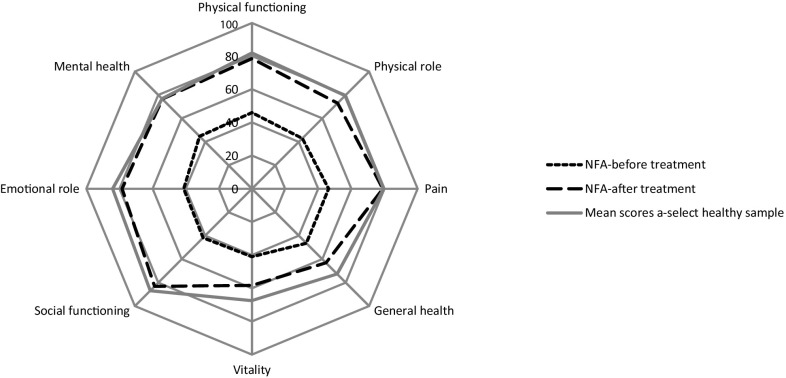
HR-QoL scores of patients with a NFA (Short Form 36 scores), figure derived from [4]. Higher scores indicate better HR-QoL

Pituitary adenomas are benign tumours, with an estimated prevalence of 78–94 cases per 100,000 individuals, and an incidence of four cases per 100,000 individuals [1]. Ten percent of all pituitary adenomas are non-functional adenomas (NFAs) [2]. NFAs commonly occur during adulthood with a median age at diagnosis of 51.5 years (range 19–79 years) [3]. At time of diagnosis, tumour size is relatively large compared to functioning tumours, since hormone excesses are absent, and therefore mainly manifest via compression of surrounding tissues, predominantly compression on the optic chiasm. Primary treatment consists of surgical resection of the tumour to relieve mass effects. Conventional radiotherapy may be used in case of tumour growth or when surgical resection is not an option due to the localization [2]. After treatment, patient reported health-related quality of life (HR-QoL) improves considerably (Fig. 1) [4], however, the evidence about normalization of HR-QoL is inconclusive. While some researchers described a persistent decreased HR-QoL compared to healthy controls and reference data [5, 6], others did not [7, 8].

Furthermore, the cause of the persistent impairments in HR-QoL seems to be multifactorial and several contributing factors have been reported, including visual function, type of surgery (craniotomy vs. transsphenoidal), hypopituitarism, and the need for hormone replacement therapy [4].

The aim of the present review was to provide an overview of health outcomes of patients with a NFA using a conceptual HR-QoL model i.e. the Wilson and Cleary model [9]. A concrete conceptualization of the health outcomes of patients with a NFA will be helpful in the understanding of the observed variety in HR-QoL outcomes, the identification of potential interventions, and can be used for further improvement of the clinical care trajectory and somatic and psychosocial guidance of these patients.

## Health-related quality of life

Over the past decade, alongside the improved treatment options, the scope of relevant outcomes has expanded from primary outcomes, such as mortality and morbidity, towards the evaluation of functional status and HR-QoL. Although it is established that HR-QoL should cover physical-, psychological-, and social well-being (in accordance with the biopsychosocial model) [10], a single concrete definition of HR-QoL is lacking, which results in major challenges for the evaluation and interpretation of HR-QoL [11]. A commonly used definition is that HR-QoL is “the functional effect of an illness and its consequent therapy upon a patient, as perceived by the patient” [12]. For the assessment of HR-QoL several measures have been developed and validated, and it is recommended that a generic measure (covering general HR-QoL domains) is combined with a disease-specific measure (covering HR-QoL aspects relevant for a specific disease) [11]. Unfortunately, a disease-specific HR-QoL questionnaire for NFA is currently lacking.

## The Wilson–Cleary model of HR-QoL

**Fig. 2:**
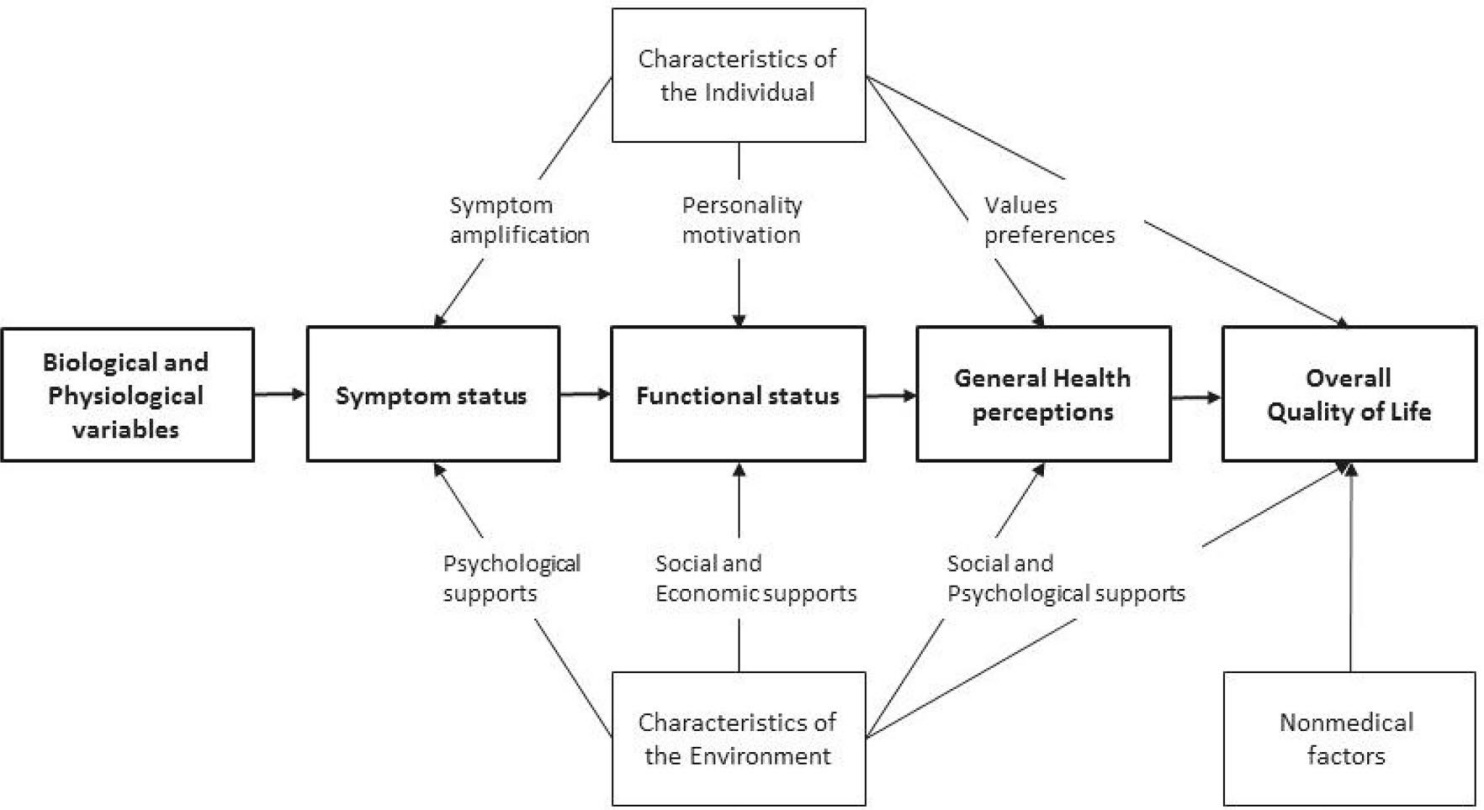
Wilson–Cleary model of HR-QoL [9]. Biological and physiological variables: function of cell, organs, and organ systems e.g. diagnoses, laboratory values, measures of physiological function, and physical examination findings. Symptom status: a patient’s perception of an abnormal physical, emotional or cognitive state. Functional status: ability of the individual to perform particular tasks. The main domains of functioning are physical functioning, social functioning, role function, and psychological function. General health perceptions: subjective rating of health, and represent and integrates all the previous health concepts

A model that is frequently used to conceptualize HR-QoL, which validity is supported by empirical evidence over the years [13], and has been widely applied to different patient populations [14–16], is the conceptual model proposed by Wilson and Cleary (1995) [9]. This model establishes the biopsychosocial model [10] by integrating the clinical paradigm (i.e. the biomedical paradigm), and the quality of life model (i.e. social science paradigm). Where the biomedical paradigm focusses on biological, physiological, and clinical outcomes, the social science paradigm focusses on dimensions of functioning and overall well-being. The Wilson and Cleary model states that health can be considered as a continuum of increasing biological, psychological and social complexity, with pure biological measures on the left side of the model, and measures of general health perceptions on the right (Fig. 2). It clarifies the proposed dominant causal relationships (bold) and mediating factors. From left to right, it goes from cell-level to the individual, to the interaction of the individual in its social context. The arrows used in Fig. 2 do not imply that there are no reciprocal relations, just as the absence of arrows does not imply that there are no such relationships. Furthermore, it should be noted that the relation between symptom status and biological and physiological variables is rather complex. In other words, biological and physiological variables can be profoundly abnormal without the patient perceiving symptoms, or the other way around. In the next paragraphs the Wilson and Cleary model will be elaborated for patients with a NFA.

## Biological and physiological variables

Pituitary dysfunction may occur in all pituitary adenomas due to a variety of causes e.g. mass effect of the tumour, surgical treatment, or radiotherapy. Severe hormone deficits, (pan)hypopituitarism, is diagnosed by blood sampling for gonadotropin, thyroid stimulating hormone, and prolactin, and dynamic stimulation tests for adrenocorticotrope hormone (ACTH), cortisol and growth hormone, and measurement of urine production for vasopressin deficiency [17]. Mild hypopituitarism can be difficult to diagnose, due to individual set-points, hormone sensitivity, and circadian variability. Nevertheless, also mild hypopituitarism may affect end organ function. Therefore, the majority of the patients with hypopituitarism need lifelong hormone replacement therapy, aiming to mimic the physiology of end organ hormones as good as possible. Replacement therapy for adrenal insufficiency is of particular relevance, since too low cortisol levels can lead to a potentially life threatening acute adrenal crisis (i.e. Addison’s crisis). Contrary to this, when replacement therapy exceeds supra-physiological cortisol levels, it can result in Cushing’s syndrome like symptoms. Therefore, adequate replacement therapy in adrenal insufficiency as well as, adaptation of the dose during stress, is crucial [18]. In clinical practice, endocrine diseases are followed by evaluating clinical signs and hormone measurements. Serum, plasma, salivary, or urinary hormone concentrations are currently the best tools for clinicians to classify disease status in (chronic) care. It has been acknowledged that the currently available physiological measures do not always reliably represent the clinical situation. The assessment of cortisol levels in scalp hair is a relatively new method to assess cortisol exposure over longer time periods and has been evaluated in patients with primary and secondary adrenal insufficiency [19]. Furthermore, it was examined whether hair cortisol levels correlated with patient reported HR-QoL, and it appeared that HR-QoL correlates slightly with hair cortisol levels [20]. These results are not surprising, considering the Wilson–Cleary model with biological and physiological variables on the one end, and HR-QoL on the other end with patient- and environmental characteristics influencing this continuum. These observations support the idea that HR-QoL is not only determined by biological disease status, but by a multidimensional underlying mechanism.

## Symptom status

When changes in biological and physiological variables occur, an individual might perceive this via symptoms. Symptom status is defined by Wilson and Cleary as a patient’s perception of an abnormal physical, emotional, or cognitive state [9]. As was mentioned previously, NFAs are usually relatively large at time of diagnosis, giving either compression on the pituitary or the optic chiasm, resulting in headaches, hypopituitarism, visual loss, third nerve palsy, pituitary apoplexy, tiredness, decreased libido, and sometimes even galactorrhoea [3]. These symptoms tend to improve after surgery, however, extensive longitudinal literature of perioperative HR-QoL is limited. Wolf et al. demonstrated that headache severity and vision related HR-QoL improved significantly up to 6 months after transsphenoidal surgery [21]. Furthermore, patients may suffer from impaired olfactory function as a complication of the transsphenoidal surgery. Little et al. showed an initial decrease of sinonasal HR-QoL after (both microscopic and endoscopic) surgery, which improved at later follow-up [22]. Wang et al. demonstrated a decrease in the ability to detect odours up to 4 months after surgery [23]. Although symptoms improve after biomedical treatment, persistent symptoms are reported after long-term remission. During focus group conversations with patients in a chronic state of their disease, patients reported physical pain, sleeping problems, changes in physical appearance (i.e. weight changes), cognitive problems (i.e. problems in concentration, short-term memory, and executive functioning), decreased libido, physical sexual dysfunction, depressive symptoms, melancholy, mood swings, worries, increased sensitivity to stress, fear of tumour recurrence, decreased self-esteem, loneliness, anger, difficulties in communication about the disease, and a lack of empathy from the environment. The reported sleep problems were characterized by sleeping in blocks of 2–3 h [24]. Sleep characteristics have also been quantitatively examined, showing sleep alterations in patients treated for a NFA, including decreased subjective sleep quality, disturbed distribution of sleep stages and disturbances in diurnal rhythmicity [6, 25]. Although it can be postulated that these sleeping problems can be explained by imperfections in hormone replacement therapy (i.e. hydrocortisone replacement) [26], there is increasing evidence that these problems are caused by hypothalamic dysfunction [27]. Joustra et al. examined sleep characteristics in patients treated for a NFA and patients with primary adrenal insufficiency treated with hydrocortisone replacement therapy and demonstrated that patients with primary adrenal insufficiency have normal sleep characteristics in contrast to patients with a NFA. These results provided evidence that sleeping problems might be caused by hypothalamic dysfunction [28]. Furthermore, sleep disturbances and daytime sleepiness were also associated with increased impairment in HR-QoL [6, 29].

## Functional status

This refers to the ability of the patient to perform particular defined tasks [9]. The symptom status largely determines whether patients perceive issues in their functioning. In accordance, the previously described symptoms result into impairments in several functional domains. During the focus group conversations patients reported problems in physical functioning, cognitive functioning, sexual functioning, psychological functioning, and social functioning. For instance, work related problems, such as diminished ability to function, to cooperate and to concentrate. As a result patients lost their job or were (partly) rejected [24]. The cognitive complaints reported by patients have also been examined through neuropsychological tests. Previous studies demonstrated that patients treated for NFA had a worse performance on verbal memory and executive functioning compared to healthy matched controls and references values [30, 31]. Interestingly, some reported the negative effect of additional radiotherapy on cognitive functioning [17, 30, 32], while others did not [33, 34].

## Characteristics of the individual

These individual characteristics (or patient characteristics) as formulated in the Wilson–Cleary model cover factors such as personality, motivation, values, and preferences. Patients’ preferences or values refer to the value patients attach to a particular consequence of their disease. For instance, a patient can experience a symptom as a burden, while the same symptom does not bother another patient. The way patients perceive their illness and its treatment are also known as ‘illness perceptions’ and ‘beliefs about medication’.

### Illness perceptions and beliefs about medication

Illness perceptions and beliefs about medication are formulated by the extended Common-Sense Model of Self-Regulation (CSM) and can be categorised into values and preference in the Wilson–Cleary model. These preferences and values play an important role at several points of the Wilson–Cleary model and are particularly important in the understanding of general health perceptions and overall HR-QoL, which is in accordance with the extended CSM, since this model also states that illness perceptions and beliefs about medication correlate with HR-QoL [35]. During the focus group conversations patients reported negative illness perceptions, such as the chronic time course of their disease, and concerns about potential side effects of their medication (i.e. hydrocortisone) [24].

### Coping strategies

Furthermore, following the extended CSM, illness perceptions and beliefs about medicines influence coping behaviour. During the focus group conversations patients reported less efficient coping strategies, such as withdrawal, and overdoing activities [24]. Coping strategies were also quantitatively assessed by Tiemensma et al. as they demonstrated that patients with pituitary disease use less effective coping strategies compared to an a-select sample of the Dutch population, including performing less active coping, seeking less social support, and using more avoidant coping strategies [36].

### Personality

A changed personality, another characteristic of the individual, is considered a problem by patients. Sievers et al. quantitatively examined personality traits in patients with a NFA, and demonstrated that compared to a normal population control group, patients with a NFA reported more neuroticism, social desirability, anticipatory worries, pessimism, fear of uncertainty, fatigability, and asthenia [37]. Individual demographic characteristics have also been found to play a role in HR-QoL in patients with a NFA, since female sex and older age were found to negatively influence HR-QoL [5, 7, 33].

## Characteristics of the environment

Economical-, psychological-, and social support, are environmental characteristics. The latter two play an important role in general health perception and overall quality of life.

During the focus group conversations patients reported unmet needs regarding care and guidance they had perceived. For instance, they would have received more information about adverse effects of medication, physical-, psychological-, and cognitive complaints and issues regarding sexual functioning. Furthermore, they would have preferred more recognition for certain complaints. These unmet needs can be categorised into characteristics of the individual (i.e. patient characteristics), since they can be influenced by personal factors. On the other hand, unmet needs can also be influenced by characteristics of the environment (e.g. availability of healthcare facilities). For example, patients reported dissatisfaction with other aspects of medical care i.e. stress-management training, lifestyle recommendations, physiotherapists, dietitians, medical sports experts, and psychologists. For instance, these unmet needs can be caused by limitations in economic support or inadequate referrals. Some types of support (e.g. psychological-, social support) are less well developed for a rare disease such as pituitary disease compared to more prevalent (chronic) diseases.

### Tools to meet unmet healthcare needs

Recently, a disease-specific patient reported outcome measure (PROM) was developed and validated by our research group, that assesses to which extent patients with pituitary disease are bothered by certain complaints, as well as their needs for support from healthcare professionals, i.e. the *Leiden Bother and Needs Questionnaire for Pituitary disease *(*LBNQ-Pituitary)*). This PROM covers five subscales i.e. mood problems, negative illness perceptions, issues in sexual functioning, physical and cognitive complaints, issues in social functioning [38]. This PROM can help healthcare professionals to address the unmet needs experienced by patients.

Besides professional environmental factors (i.e. healthcare facilities), there are also personal environmental factors. Often the single most important person in a patient’s social network is their spouse or partner. Focus group conversations with partners of patients with a pituitary condition revealed that partners were worried about the complaints of the pituitary disease, had negative beliefs about medication, and perceived coping challenges, relationship issues, social issues, and unmet needs regarding care [39]. These observations clearly demonstrate that chronic care for patients with pituitary disease is not limited to just the patient. In order to support patients and their partners in coping with the consequences of the pituitary disease, a self-management program was developed for patients with pituitary disease and their potential partners i.e. *Patient and Partner Education Program for Pituitary disease* (*PPEP-Pituitary*). This program aims to (at least partly) fulfil the unmet needs regarding support for psychological and social issues. PPEP-Pituitary was based on a standardized Patient and Partner Education Program initially developed for patients (and partners) with Parkinson’s disease [40]. A multicenter randomized-controlled trial revealed that patients reported more self-efficacy after PPEP-Pituitary which was still present after 6 months. Self-efficacy is described in the ‘Social Cognitive Theory’ of Bandura [41] and is defined as the person’s beliefs in his or her own capabilities and skills to perform a certain action, in a certain situation. Following this theory, behavior is directly influenced by goals and self-efficacy beliefs. In accordance, several studies showed that self-efficacy beliefs influences self-management behavior [42, 43]. Patients also reported to be less bothered by mood problems directly after PPEP-Pituitary, however this returned to baseline levels 6 months later. Partners reported an increase in vitality, a decrease in depressive symptoms and an increase in treatment control after PPEP-Pituitary. This persisted at follow up after 6 months [44]. It can be postulated that offering this program as standard clinical care will improve the quality of the (healthcare) environment, and ultimately the patient and partner reported HR-QoL.

## General health perceptions and HR-QoL

In accordance to the Wilson–Cleary model, the domains described in the preceding paragraphs all contribute to patient perceived HR-QoL. This increase in interest in HR-QoL has led to an increase in the number of HR-QoL studies in patients with a NFA and these studies show some diversity regarding HR-QoL outcomes. Johnson and colleagues reported HR-QoL impairments in patients with an untreated NFA, especially in physical and mental functioning during active disease [45]. Some confirmed this decreased HR-QoL in patients treated for a NFA compared to reference values and healthy controls [5, 6], however others did not report any differences [7, 8, 46]. Furthermore, some studies demonstrated the negative effect of tumour recurrence [7], hypopituitarism [5, 47] and radiotherapy [48] on HR-QoL, while other did not (pituitary deficiency [46], radiotherapy [33, 46]). In addition, no differences in HR-QoL were found between patients surgically treated for a NFA and patients treated with mastoid surgery [48]. No differences were found while comparing patients with growth hormone deficiency (GHD) due to a NFA compared to traumatic brain injury [49, 50]. Male patients with GHD due to a NFA, compared to patients with GHD due to a craniopharyngioma, reported a better HR-QoL, whereas female patients with a NFA reported a worse HR-QoL [51]. Intervention studies reported that HR-QoL of patients with a NFA improved after transsphenoidal surgery [47, 52]. Furthermore, patients treated with craniotomy reported more HR-QoL impairments compared to patients treated with transsphenoidal surgery [8]. There have been several systematic reviews on endoscopic and microscopic transsphenoidal surgery, describing comparable or better clinical results after endocopic surgery [53]. Furthermore, a qualitative study performed by Lwu et al. described that patients perceived less burden after endoscopic surgery compared to microscopic surgery [54]. However, there is limited knowledge about the long-term outcomes of endoscopic vs. microscopic surgery in terms of HR-QoL. Intervention studies about the effect of growth hormone replacement therapy in patients treated for NFA with GHD all reported a positive effect on HR-QoL [49–51, 55–57]. On the other hand, a cross-sectional study of Capatina et al. demonstrated that non-replaced GHD was an independent predictor of a better score in bodily pain, general health perception and energy/vitality [7].

## Conclusion

**Fig. 3:**
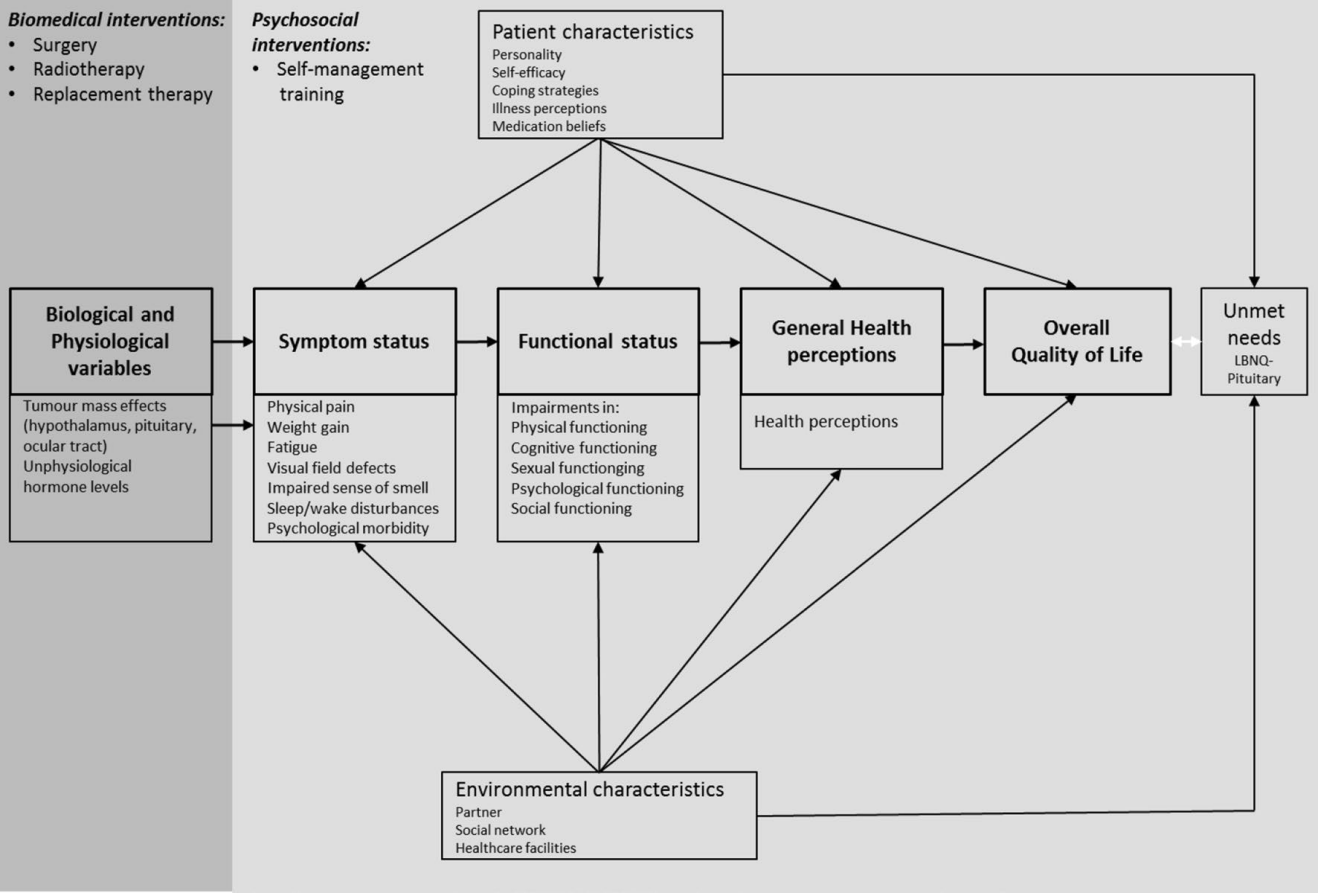
Wilson–Cleary model of HR-QoL elaborated for NFA

The present review emphasizes that although patients may be in a stable medical condition, health issues are present at each level of the Wilson–Cleary model (Fig. 3). Application of the Wilson–Cleary model to patients with a NFA enables to observe that persistent impairments in HR-QoL might be explained by issues at each stage of this model. This also provides further insight into why there is such a variety in clinical outcomes, and why some patients experience severe problems, while others experience either no or only mild problems. This emphasises that improvement in overall HR-QoL in patients with pituitary disease requires optimal biomedical treatment initiating a cascade of improvement in health outcomes starting with a better symptom status. Nevertheless, this model also clarifies that besides the currently available biomedical interventions (i.e. surgery, radiotherapy, hormone replacement therapy) targeting biological and physiological variables, interventions are needed that pay attention to other (psychosocial) elements of the model e.g. cognitive functioning, sexuality/intimacy, psychological well-being, social functioning, coping behaviour, self-efficacy beliefs, illness perceptions, medication beliefs, quality of the partner relationship, and the social network/support. Therefore, further improvement of HR-QoL should be supported by a pituitary specific care trajectory, including psychosocial care (e.g. self-management training), in order to beneficially affect characteristics of the patient and the (healthcare) environment, with the utmost goal to optimize HR-QoL in patients after treatment for a NFA.
